# Hybrid Encryption Algorithm Based on Gray Curve and Josephus Permutation

**DOI:** 10.1155/2022/7076416

**Published:** 2022-10-26

**Authors:** Ying Niu, Hangyu Zhou, Xuncai Zhang, Limin Qin

**Affiliations:** ^1^School of Architecture Environment Engineering, Zhengzhou University of Light Industry, Zhengzhou 450002, China; ^2^School of Electrical and Information Engineering, Zhengzhou University of Light Industry, Zhengzhou 450002, China; ^3^Henan Topnet Computer Engineering Company Limited, Zhengzhou 450000, China

## Abstract

This paper proposes a new image encryption algorithm based on chaos map systems, hash algorithm, and Josephus permutation. The algorithm consists of chaos initialization, pixel position permutation, and pixel information diffusion. The algorithm's initialization is generated by the original image, which has a high sensitivity to the initial value. The permutation step length is composed of Josephus permutation and gray curve permutation, which completely disturbs the pixel distribution. The diffusion process is composed of cross operation and ciphertext feedback, which breaks the strong correlation between pixels. The simulation results of the encryption algorithm are used to analyze its information entropy, the correlation between elements, and other indicators. The ciphertext image is attacked in several ways, and we analyzed its defense ability. Simulation results show that the algorithm can effectively encrypt image information and has a good defense against various attacks.

## 1. Introduction

In recent times, with the rapid development of science and technology, information and data security play an increasingly important role. To protect the important information of private, enterprise, and government, how to improve the security performance of image transmission is an urgent problem to be solved. To ensure the integrity and security of information, many encryption algorithms are used to protect the security of network information, such as DES [1] and AES [2]. Shannon [3] laid a solid foundation for cryptography and encryption systems and put forward the famous encryption system theory, and many encryption algorithms came into being. Mishra and Mankar [4] proposes a text encryption algorithm using pseudorandom number generator and nonlinear function. Babaei [5] proposes an encryption algorithm based on DNA computing, which can realize parallel encryption of a large number of data. L. D. Singh and K. M. Singh [6] proposes a new encryption algorithm based on elliptic curve cryptography, which provides higher security performance with smaller key lengths and reduces the high cost of the map operation.

Because the image information has the characteristics of a strong correlation between adjacent elements, when the image information is transmitted, the existing encryption algorithm is unable to meet the increasing demand for encryption performance. Therefore, an algorithm with stronger encryption performance is needed to encrypt and protect the image information. With the development of chaos theory, more and more scholars turn their attention to the encryption algorithm based on the chaos system. The encryption algorithm based on chaotic is more suitable for image encryption because of its ergodicity, high sensitivity, and pseudorandom characteristics. But it would be too simplistic to encrypt images with only chaotic sequences. Scholars began to combine the Josephus problem with chaotic sequences to encrypt images. The Josephs traversal method selects the step size by using the pseudorandom sequence generated by the chaotic system and selects the corresponding image pixels cyclically to achieve the effect of scrambling. Many encryption algorithms use Josephus traversal to realize image encryption. Xu et al. [7] proposed an image encryption scheme based on the combination of Josephus permutation and image filtering. Adjacent pixels can be quickly scattered into different rows and columns. To achieve the purpose of encrypting the image, Wang et al. [8] proposed an image encryption scheme based on Josephus traversal and mixed chaotic maps. The image is encrypted by scrambling the pixel position using Josephus traversal and then using the position to randomly change the pixel value. Hua et al. [9] proposed an image encryption algorithm utilizing the principles of the Josephus problem and the filtering technology. The chaotic sequence can also be used to scramble the bit-level of the pixel. Gan et al. [10] introduces a novel chaos-based image encryption algorithm for color images based on three-dimensional (3-D) bit plane permutation. Wu et al. [11] proposed a new color image encryption algorithm combined with rectangular transformation. The algorithm can encrypt three primary color channels of the image at the same time.

With the continuous improvement of computing power and information security requirements under the Internet background, image encryption algorithm needs to have a more complex structure and higher sensitivity to be more difficult to crack. The break of the MD5 function [12] and the update of computer hardware are more and more threatening to the encryption system. Image encryption technology can be roughly divided into two categories at present: compressed image encryption technology and spatial image encryption technology. The use of compressed sensing technology to encrypt images has developed rapidly. The image is encrypted and compressed at the same time, and then embedded into the carrier image to achieve the purpose of encrypting the image. Hua et al. [13] proposed a visually secure image encryption method based on adaptive threshold sparsification and parallel compressed sensing. The algorithm can greatly improve the quality of reconstructed images and has higher encryption efficiency and security. The spatial image encryption technology is used to encrypt the uncompressed image. That is, the image is operated as a two-dimensional matrix. Spatial image encryption technology is divided into two types: symmetric encryption and asymmetric encryption. At present, the most commonly used encryption method is the “obfuscation-diffusion” structure, which is also a symmetric key encryption scheme. A chaotic system with a weak structure is more vulnerable to attack and break, resulting in information loss and leakage. The two-dimensional chaotic system has more parameters, a more complex structure, better ergodicity, sensitivity, and other characteristics. So the encryption algorithm based on two-dimensional chaotic system usually has a large key space and strong security performance. Hua et al. [14] proposed a color image encryption scheme based on the combination of orthogonal Latin squares and a new 2D chaotic system. First, the pixels of the two-dimensional matrix are scrambled by using Latin squares, and then the pixel-level diffusion is performed on the scrambled image. This method can effectively solve the relationship between color images and Latin squares. Chai et al. [15] presents a color image cryptosystem based on dynamic DNA encryption and chaos. Based on the logistic map, Jin et al. [16] proposed an encryption algorithm, which combines the pre-encrypted image with the original gray value in disorder. Tong and Liu [17] proposed an encryption algorithm based on high-dimensional dynamic multiple chaotic maps, which combined with cyclic displacement to produce a faster avalanche effect and excellent encryption effect. Som et al. [18] proposed a symmetric key encryption algorithm for color images based on chaotic map and pseudorandom binary number generator (PRBNG), which has a very large key space to improve the security of the encryption system. Hua et al. [19] proposed an image encryption scheme for the S-box of complete Latin squares. S-box is the nonlinear part of a symmetric key encryption scheme. It directly determines the performance and security level of the encryption scheme. This paper applies S-box to image encryption applications. The result of the analysis shows that it has high security and can effectively resist differential attacks, antilinear attacks, and other attacks. Zhang and Wang [20] proposed a new spatiotemporal chaotic image encryption algorithm based on the hybrid linear-nonlinear coupled chaotic map. The bit-level pixel arrangement strategy makes the low-bit plane and the higher-bit plane of the pixel displace each other without any additional storage space, greatly improving the calculation speed and reducing the calculation cost. Norouzi et al. [21] proposed an encryption algorithm based on a hyperchaos system, which combines the masking method and bit plane diffusion, and achieves high security of the encryption system.

To further enhance the chaotic characteristics of the encryption algorithm and enhance the defense ability against the attack, a variety of coupled chaotic maps have a better prospect. This paper proposes an efficient encryption scheme for image data. The algorithm is based on the pseudorandom sequence generated by PWLCM map and 2D-LSCM map, combined with gray curve scrambling, Josephus traversal, and other encryption methods, to achieve effective encryption and protection of image data. The second section of this paper introduces the theoretical basis of generating pseudorandom sequences and encryption methods in the proposed algorithm. The third section introduces the specific calculation steps to realize image encryption. The fourth section analyzes the security of various data and the ability to resist various attacks on the experimental simulation results, and the results show that the algorithm has fast calculation, strong security, and good defensive performance. Therefore, the encryption system can be used for digital image encryption.

## 2. Basic Theory

### 2.1. Piecewise Linear Map

In the algorithm proposed in this paper, we use piecewise linear map to generate pseudorandom sequences needed by Josephus traversal. Piecewise linear map is PWLCM [22] for short, and its definition is shown in the following formula:

(1) $$\begin{array}{c} x_{i}=F\left(x_{i-1},\tau \right)=\left\{\begin{array}{c} \frac{x_{i-1}}{\tau },x_{i-1}\in \left.\left[0,\tau \right.\right)\\ \left.\frac{x_{i-1}-\tau }{0.5-\tau },x_{i-1}\in \left(\tau \right.,0.5\right]\\ F\left(1-x_{i-1},\tau \right),x_{i-1}\in \left(0.5,1\right) \end{array}\right.\text{,} \end{array}$$

where *F*(*x*_*i*−1_, *τ*) is PWLCM map, *x* ∈ [0,1), and control parameter *τ* ∈ (0,0.5). The chaotic system can enter a stable chaotic state and the control parameter *τ* is a part of the key.

**Figure 1:**
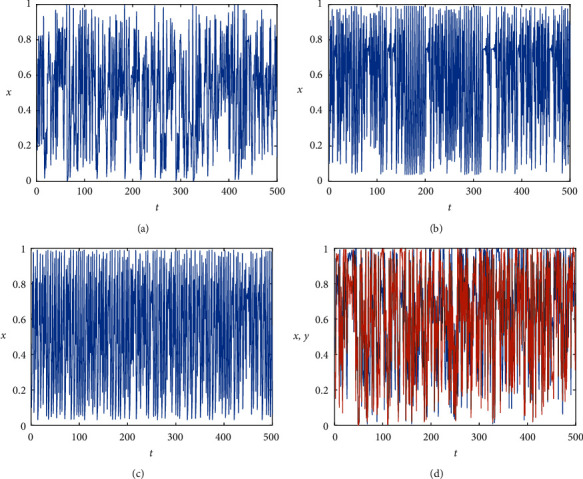
Simulation diagrams. (a) PWLCM map. (b) Logistic map. (c) Sine map. (d) 2D-LSCM map.

**Figure 2:**
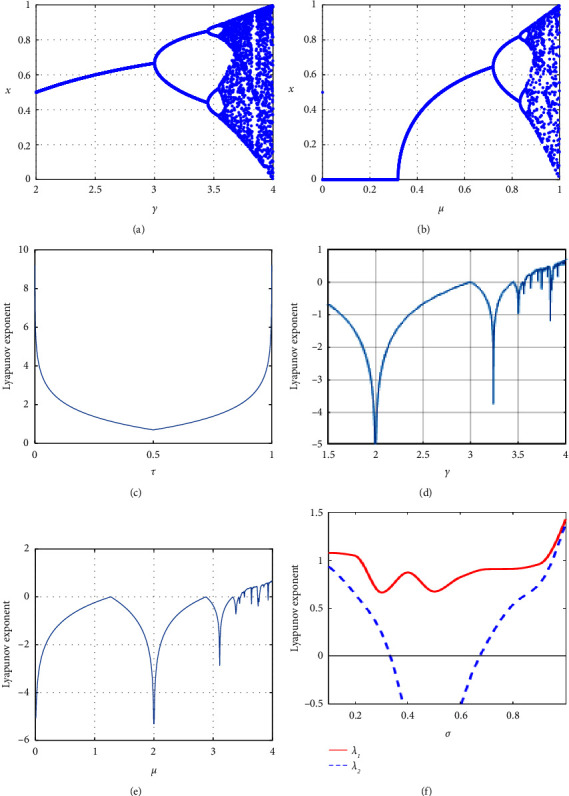
Bifurcation and Lyapunov exponent diagrams. (a) Bifurcation diagram of logistic mapping. (b) Bifurcation diagram of sine mapping. (c) Lyapunov exponent of PWLCM mapping. (d) Lyapunov exponent of logistic mapping. (e) Lyapunov exponent of sine mapping. (f) Lyapunov exponent of 2D-LSCM mapping.

The simulation diagram of the PWLCM chaotic system is shown in Figure 1 and the Lyapunov exponential diagram is shown in Figure 2. It can be seen that the PWLCM chaotic system has good pseudorandomness and ergodicity. PWLCM chaotic system is a one-dimensional chaotic system. Although its structure is relatively simple, its time and space complexity is relatively small. When generating pseudorandom sequences in the proposed encryption system, it can provide high efficiency and save calculation costs. We choose the sequence generated by the PWLCM system as the step length of the Josephus traversal. The ergodicity and robustness of the PWLCM system are enough to meet the requirements of Josephus traversal.

### 2.2. 2D-LSCM Map

The two-dimensional logistic sine coupled map (2D-LSCM) is a coupling of one-dimensional logistic map [23, 24] and sine map [25]. The definition of logistic map is shown in the following formula:

(2) $$\begin{array}{c} x_{i+1}=4\gamma x_{i}\left(1-x_{i}\right)\text{.} \end{array}$$

The control parameter *γ* ∈ (0,1). The definition of sine map is shown in the following formula:

(3) $$\begin{array}{c} x_{i+1}=\mu \sin\;\left(\pi x_{i}\right)\text{.} \end{array}$$

The control parameter *μ* ∈ (0,1).

The bifurcation and simulation diagrams of logistic map and sine map are shown in Figures 1 and 2. It can be concluded that the security of the encryption algorithm is not high due to the lack of ergodicity and other reasons when using logistic map or sine map for the encryption system alone [26]. Therefore, to obtain the pseudorandom sequence, which is more suitable for the encryption algorithm, Hua coupled the logistic map and the sine map to get a new chaotic map, which is called 2D-LSCM map [27]. It is defined as follows:

(4) $$\begin{array}{c} \left\{\begin{array}{c} x_{i+1}=\sin\left(\pi \left(4\sigma x_{i}\left(1-x_{i}\right)+\left(1-\sigma \right)\sin\;\left(\pi y_{i}\right)\right)\right)\text{,}\\ y_{i+1}=\sin\left(\pi \left(4\sigma y_{i}\left(1-y_{i}\right)+\left(1-\sigma \right)\sin\;\left(\pi x_{i+1}\right)\right)\right)\text{.} \end{array}\right. \end{array}$$

The control parameter *σ* ∈ (0,1). The simulation diagram of 2D-LSCM map is shown in Figure 1. In Figure 1, the lines of different colors represent the motion tracks of different variables. The coupled two-dimensional chaotic map significantly improves the characteristics of chaotic system, which has strong ergodicity and sensitivity, and can provide greater key space and higher security for the encryption algorithm. Figure 2 shows the Lyapunov exponential diagram of the 2D-LSCM map. A positive Lyapunov exponent means the map is chaotic and a larger LE means better chaotic behaviors [28, 29]. Besides, the diagram shows the 2D-LSCM map has more than one positive LEs, which has extremely good chaotic behavior. Therefore, 2D-LSCM map has a wider chaotic range and more complex chaotic behavior.

### 2.3. Gray Curve

**Figure 3:**
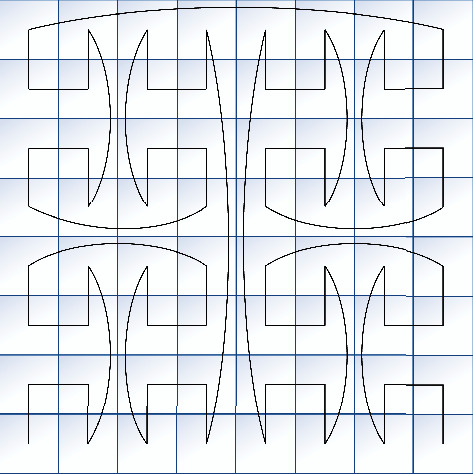
Gray curve.

Fractal geometry is an assumption proposed by Mandelbrot, which is based on many natural phenomena with self-similarity and high repetition. Fractal geometry is different from Euclidean geometry. It is irregular, but it has a scale structure. The fractal structure can be observed on every scale. Fractal geometry has the characteristics of self-similarity, highly complex structure, high repeatability, and iteration. General fractal sets include Mandelbrot set, Julia set, Koch curve, Cantor set, and Sierpinski set. Fractal images can be used in the permutation operation of image encryption [30, 31] and get a better confusion effect. The gray curve is generated by the evolution of gray code proposed by Baudot. Because only one of its adjacent bits changes, the logic confusion caused by the change between two states is greatly reduced. The pulse interference generated in the digital circuit is avoided and the error generation is minimized during the digital-to-analog conversion. The gray curve is one of the fractal sets, and its representation is shown in Figure 3.

The gray curve evolved from gray code, and its iteration rules are shown in Table 1. Firstly, a set of one-dimensional gray codes with values of 0 and 1 is generated. Then two-dimensional gray codes are generated based on one-dimensional gray codes, whose values are 00, 01, 11, and 10. That is to say, when generating the (*n* *+* 1)-dimensional sequence, the first 2^*n*^ elements are all elements of the *n*^th^ dimension sequence in order, and the prefix 0 is added; the next 2^*n*^ elements are all elements of the *n*^th^ dimensional sequence in reverse order, and the prefix 1 is added. Using computer simulation to generate gray code, which can be obtained in the following ways: keeping the highest bit of the natural binary number to be converted and taking it as the highest bit of gray code; performing exclusive OR operation on the highest bit and the secondary high bit of binary code and taking the result as the secondary high bit of gray code; calculating the remaining bits in the same way. Set the binary number to be converted to *X*_*n*_*X*_*n*−1_*X*_*n*−2_,…, *X*_1_ and the code is *Y*_*n*_*Y*_*n*−1_*Y*_*n*−2_,…, *Y*_1_ by using the formula (5) to convert

(5) $$\begin{array}{c} Y_{i}=xor\left(X_{i},X_{i-1}\right)\text{,} \end{array}$$

where *i*=1,2,…, *n* − 1 and XOR is exclusive or operation. After getting the n-dimensional gray code, we can get the gray curve based on the code: convert the *X* coordinates and *Y* coordinates of image pixels into binary values corresponding to their coordinate values, and calculate their corresponding gray codes; cross the coding of the *X* coordinates and the *Y* coordinates and calculate the gray code based on the new coding, and then connect the pixel positions of these coding from small to large. Then we get the corresponding gray curve.

**Figure 4:**
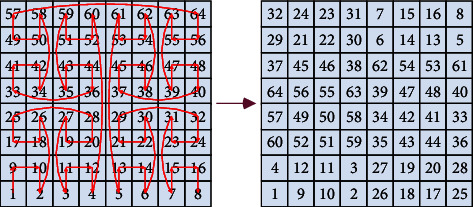
8*∗*8 image matrix gray permutation.

The function of the gray curve in the encryption system is to take out the pixels of different positions through gray coding transformation to disorder the gray value distribution. The gray permutation effect of the 8*∗*8 image is shown in Figure 4. According to the direction of the curve, the pixels are taken out and arranged in order, and then recombined into a matrix.

## 3. Encryption Scheme

### 3.1. Chaos Initialization

To make the system highly sensitive to the key and the initial image, the control parameters of the chaotic system are calculated by using the variable *q*_*i*_ related to the gray value of the plaintext image pixels. Suppose that the gray value of the plaintext image pixel is *P*_*i*,*j*_, where *i* ∈ (0, *M*), *j* ∈ (0, *N*), the control parameters *σ* and *τ* of the chaos map are calculated according to the following formula:

(6) q1=4MN∑i=1M2∑j=1M2modPi,j∗10 ^ 6,256, q2=4MN∑i=M2+1M∑j=1M2modPi,j∗10 ^ 6,256,q3=4MN∑i=1M2∑j=N2+1NmodPi,j∗10 ^ 6,256,q4=4MN∑M2+1M∑N2+1Nmod Pi,j∗10 ^ 6,256,τ=1−cos mod q1+q22,2π3−π3,σ=12sinmod q3+q42,π2+π2, 

where *M*, *N* is the image specification, *P*_*i*,*j*_ is the gray value of the corresponding position of the pixel matrix, *q*_1_, *q*_2_, *q*_3_, and *q*_4_ is the intermediate calculation variable, *τ* is the control parameter of the piecewise linear map, and *σ* is the control parameter of the logistic sine map. Formula (6) uses the trigonometric function twice to make the control parameter in the required range of the positive Lyapunov exponent. The encryption system initializes the sequence value with the initial image. When the initial image has a small change, the encryption result of the encryption system will change greatly. Therefore, the initial chaotic system is sensitive to the initial image.

The initial value of the chaotic system is generated by the hash algorithm. Take the original image *P* as the input. The hash sequence *H* with a length of 384 bits is obtained through the calculation of the SHA-384 algorithm. Then take the first 64 bits of the sequence *H* as the value *H*_1_, and calculate *c*_1_ according to formula (7). Take *c*_1_ as the initial value of PWLCM map. Take the 65th to 128th bits of the sequence *H* as the value *H*_2_, and the 129th to 192nd bits are taken as the value *H*_3_. The values *c*_2_ and *c*_3_ are calculated according to formula (7) and are taken as the initial values of the 2D-LSCM system.

(7) $$\begin{array}{c} c_{i}=\frac{H_{i}}{1.9*10^{19}}-floor\left(\frac{H_{i}}{1.9*10^{19}}\right)+c_{i}^{0}\text{,} \end{array}$$

where *c*_*i*_^0^ is the given value, *i* *=* 1, 2, and 3. The initial value of the chaotic system is determined by the original image and the given value, so it has a large key space and high sensitivity to the original image, which can effectively resist attacks.

This article uses the hash value of the plaintext image as part of the key, and we need to assign the key to other users. We can distribute it to other users through decentralized key distribution, centralized key distribution, etc. Decentralized key distribution allows each user to be assigned a session key in a secure manner. In centralized key distribution, a key center is used to exchange session keys with users.

### 3.2. Permutation Encryption

**Figure 5:**
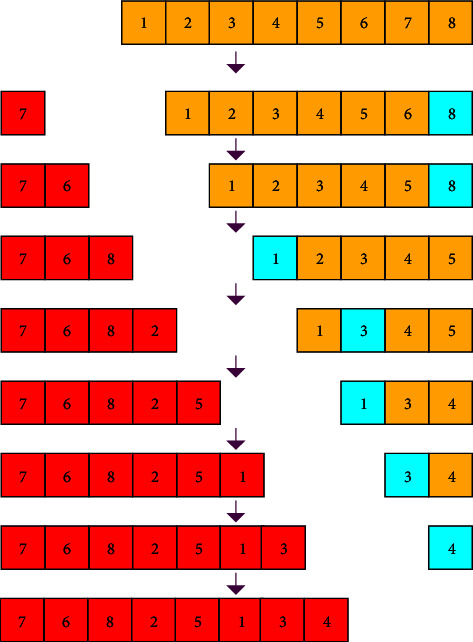
Josephus permutation effect with step length of 7.

Josephus permutation is an algorithm derived from an ancient allusion [32]. In the war against the Roman army, Josephus fled to a hiding place with a friend and 39 Jews after Jotapat was captured by Rome. These Jews would rather die than be captured. They agreed to commit suicide by way of reporting in turn. All of them were in a circle, starting from the first reporter. Every time they reported to the third person, the one had to commit suicide. And then the next one counts again until everyone commits suicide. After the Josephus problem is transformed into a mathematical problem and simplified, it can be described as a circle of several elements. Each element is checked and counted from the beginning to the end, and the kth element is taken out. Then count from the next element adjacent to the extracted element again, and loop through the above operations until the last element is removed. According to the order of being taken out, the elements are arranged in order, and we get the expected sequence of the Josephus permutation result. We perform Josephus permutation according to to the formula below:

(8) $$\begin{array}{c} f\left(x,k\right)=k\;\mathrm{mod}\left(x-i+1\right)\text{,} \end{array}$$

where *x* is the length of the element sequence involved in permutation and *k* is the sequence number of “suicide” specified in advance, which is the step length, and *i* is the order of operation. In the proposed encryption algorithm, the first number of sequence *Y* is used as the fixed step length to permute the first-row elements of the image matrix; the second number of sequence *Y* is used as the fixed step length to permute the second-row elements of the image matrix. According to this rule, the row elements of image matrix are permuted by Josephus traversal for *M* times. Next, Josephus permutation is operated in the first column of the image matrix with the (*M* *+* 1)*-*th number of sequence *Y* as the fixed step length; then permute the second column of the image matrix with the (*M* *+* 2)-th number of sequence *Y* as the fixed step length until permuting all columns of image matrix for *N* times. The Josephus permutation example with a step length of 7 for the element sequence with a length of 8 is shown in Figure 5, in which the element marked in cyan is the starting position of the next count.

In the simulation of image permutation, we find that if we use Josephus traversal with variable step length, the encryption system has weak resistance to data loss attack and noise attack. Although, when only a few pixels are changed, the decryption system will not be able to decrypt the original image correctly, and the decrypted image is noisy. If Josephus permutation with fixed step length is used, the elements in a certain gray value range will not be fully permuted. Using gray curve permutation as a supplement to Josephus permutation with fixed step length solves this problem well, and the problem of low key space of gray curve permutation is also solved. Therefore, we use these two schemes to fully confuse the location of each pixel, and the system has a strong ability to resist noise and data loss attacks.

### 3.3. Diffusion Encryption

#### 3.3.1. XOR Ciphertext Feedback

**Figure 6:**
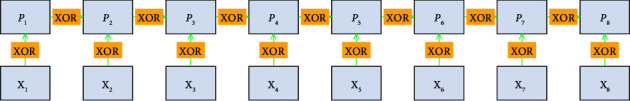
XOR ciphertext feedback.

XOR ciphertext feedback [33] is an operation to make adjacent elements in the image influence and confuse their gray values to enhance the diffusion effect of the algorithm. In a single calculation, generally, the elements in the first place will affect the elements in the second place, and the XOR ciphertext feedback process will be repeated and operated in a different order many times to ensure that all elements experience XOR ciphertext feedback as much as possible, so that the encryption scheme can achieve a better diffusion effect. The process of XOR ciphertext feedback is as follows: firstly, the image matrix to be encrypted is expanded into a sequence with a length of *M∗N*, and the *M∗N* elements in the pseudorandom sequence *Z* generated by the chaotic system are operated by bit plane according to their positions, and then each element and the next adjacent element are calculated by bit XOR according to formula (9) from the beginning to the end.

(9) $$\begin{array}{c} B\left(i\right)=bitxor\left(bitxor\left(I\left(i\right),Z\left.\left(i\right)\right),I\left(i-1\right)\right)\right)\text{,} \end{array}$$

where 2 ≤ *i* ≤ *M∗N*, *M∗N* is the specification of the image to be encrypted, *I*(*i*) is the value of each element of the one-dimensional sequence of the image to be encrypted, and *B*(*i*) is the value of each element of the obtained one-dimensional sequence of ciphertext.  Figure 6 shows a flow example of XOR ciphertext feedback. In this encryption step, the gray values of image pixels are confused with each other and the pseudorandom sequences generated by the chaotic system.

The encrypted image matrix is obtained by recombining the one-dimensional sequence obtained by XOR ciphertext feedback into *M∗N* matrix. XOR ciphertext feedback not only confuses the noise information carried by the key with the original image, but also spreads the gray values of different pixels in the image, so it is difficult for attackers to obtain information from the encrypted image.

#### 3.3.2. Bit Crossover

The crossover operation is a kind of genetic algorithm, which aims to generate new offspring by rearranging the “genetic information” of two parents according to certain new rules [34]. When this idea is applied to image encryption, it will produce the effect of confusion on the bit plane. In this algorithm, the number in the pseudorandom sequence obtained by the 2D-LSCM algorithm is used as the crossover operator after the modulo operation of 256, and the number in the encrypted image matrix is diffused under the crossover operation. The gray value of every two-parent pixel and the crossover operator are represented by an 8-bit binary number, then the value of each pair of parent bits determines the switching position or remains unchanged according to the value of the crossover operator. If the value of a bit of the crossover operator is 0, the value of the corresponding bit of the parents is exchanged, and the new individual inherits the bit values of different positions, respectively. If the value is 1, the value of the corresponding bit of the parent remains the same, and the child inherits the bit values of the same position.  Table 2 shows the algorithm principle of generating new elements through bit recombination and diffusion. For example, suppose that the two parents *A* and *B* participating in the crossover operation are 10111010 and 01000101, respectively, and the crossover operator is 10010110. The crossover results of *A*′ and *B*′ is shown in the table.

**Figure 7:**
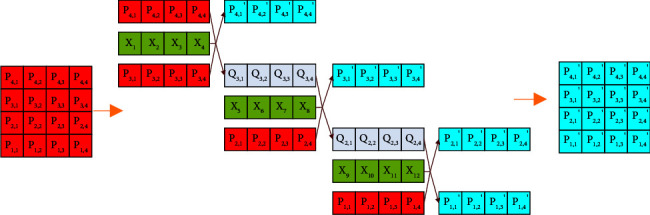
XOR crossover operation encryption.

In the encryption algorithm proposed in this paper, the first to *N*^th^ numbers in sequence *X* are used as crossover operators to perform the crossover operation and the first to *N*^th^ pairs of elements in the first and second rows are diffused. The crossover operators are *X*_1_, *X*_2_, *X*_3_,…, *X*_*N*_; use the (*N* *+* 1)^th^ to (2*N*)^th^ number in the sequence *X* as the crossover operator to perform the crossover operation on the first to *N*^th^ elements of the second and third row, and the crossover operators are *X*_*N+*1_, *X*_*N+*2_, *X*_*N+*3_,…, *X*_2*N*_; and so on, until the *M* row of the image matrix is diffused, the crossover operator is *X*_(*M* − 2)*∗N*+1_, *X*_(*M* − 2)*∗N*+2_, *X*_(*M* − 2)*∗N*+3_ … *X*_(*M* − 1)*∗N*_. The encryption rules for the cross-operation of the 4*∗*4 matrix are shown in Figure 7. Then the image matrix is operated by columns according to the same rules.

Through the crossover operation, the gray value of each pixel is confused and affects the adjacent elements, and then it spreads to the gray value of all other elements of the image through a continuous iterative operation.

### 3.4. Encryption Process

**Figure 8:**
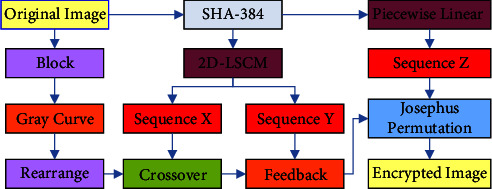
Flow chart of encryption algorithm.

Due to the high correlation or noise between pixels of image data, a good image encryption system should be able to overcome these shortcomings when it is attacked by data loss attack, statistical attack, etc. Based on the pseudorandom sequence generated by 2D-LSCM, the encryption algorithm proposed in this paper confuses the image pixel position by gray curve and Josephus permutation operations; and carries out crossover operation and exclusive or ciphertext feedback operations for gray value information of each pixel. The specific flow chart of the encryption algorithm proposed in this paper is shown in Figure 8.

The chaos is initialized as described in 3.1. The control parameters and initial values of the encryption system are generated according to the initial image. The initial values are input into PWLCM map and 2D-LSCM map respectively, and the pseudorandom sequences *SX*, *SY*, and *SZ* are obtained. The three sets of sequences were amplified and modeled according to formula 10 to obtain sequences *X*, *Y*, and *Z*.

(10) $$\begin{array}{c} \left\{\begin{array}{c} X\left(i\right)=\mathrm{mod}\;\left(SX\left(i\right)*10^{10},256\right)\text{,}\\ Y\left(i\right)=\mathrm{mod}\;\left(SY\left(i\right)*10^{10},256\right)\text{,}\\ Z\left(i\right)=\mathrm{mod}\;\left(SZ\left(i\right)*10^{10},256\right)\text{.} \end{array}\right. \end{array}$$

**Figure 9:**
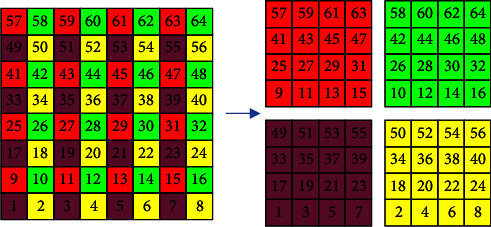
Block rule.

1. Divide the original image matrix *P* into blocks. The matrix *P* of the original image is indexed, in which element *P*(*i*, *j*) makes up matrix *K*_1_ element *P*(*i*, *j*+*N*/2) makes up matrix *K*_2_, element *P*(*i*+*M*/2*M*/2, *j*) makes up matrix *K*_3_, and element *P*(*i*+*M*/2, *j*+*N*/2)*ma*ke up matrix *K*_4_, where *i*=1, 2 …  *M*/2;  *j*=1, 2 …  *N*/2. If the number *M* of rows or the number *N* of columns is odd, a suitable number of noise matrices are added to the edge of the image so that it can be divided into four block matrices with equal specifications. The block processing method is shown in Figure 9.
2. The gray curves permutation of the pixels in the matrices *K*_1_, *K*_2_, *K*_3_, and *K*_4_ is operated, respectively, as described in Section 2.3.
3. Reassemble the scrambled four block matrices in the upper left corner, the upper right corner, the lower left corner, and the lower right corner to obtain the reconstructed image matrix *P*_1_. Take out the first (*M* − 1)*∗N* elements in the *X* sequence and take them as the crossover operator, and carry out bit crossover operation on the elements in the image matrix *P*_1_ as described in Section 3.3.2 and obtain the image matrix *P*_2_.
4. Take out the *M∗N* pseudorandom values in the sequence *Y*, and carry out bit exclusive or with the element pixel values in matrix *P*_2_ and convert the obtained matrix into a sequence and diffuse the elements by bitwise exclusive or from the beginning to the end. Repeat it twice in positive and reverse order, and then restore the sequence to the *M∗N* matrix to get matrix *P*_3_.
5. Take out *M* *+* *N* pseudorandom values in sequence *Z*, and use them as the step length of Josephus permutation. Josephus permutation operation is performed on image matrix *P*_3_ as described in Section 3.2 to obtain the final encrypted image matrix *P*_4_.

The decryption algorithm is the inverse operation of the encryption algorithm, which will not be discussed here.

## 4. Simulation Result and Security Analysis

**Figure 10:**
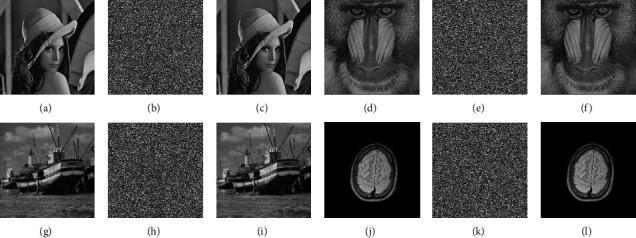
Plain, encrypted, and decrypted images. (a) Lena. (b) Encrypted Lena. (c) Decrypted lena. (d) Baboon. (e) Encrypted baboon. (f) Decrypted baboon. (g) Boat. (h) Encrypted boat. (i) Decrypted boat. (j) Brain. (k) Encrypted brain. (l) Decrypted brain.

Detecting the security performance of an image encryption algorithm is the standard to evaluate its feasibility. The analysis indexes of the encryption system are key space, histogram analysis, information entropy analysis, NPCR, and UACI. To test the encryption effect of the proposed algorithm, we have carried out simulation experiments on the encryption algorithm. The experimental environment is as follows: CPU: Intel (R) Pentium (R) g3220, 3.00 GHz; memory: 4.00 GB; operating system: Windows 7; core tool: MATLAB 2017. The encryption algorithm takes *c*_1_^0^=*c*_2_^0^=*c*_3_^0^=0.01 in security analysis. The size of 256*∗*256 plain and corresponding encrypted images and their decrypted images are shown in Figure 10. It can be seen that the algorithm can effectively encrypt the original image, and the decrypted image has no loss and will not destroy the original information contained in the file to be encrypted. Next, we test each index of the encryption algorithm.

### 4.1. Keyspace

Of all the attacks against encrypted information, the brutal attack is the most common and simple way. The attacker attempts to crack the password text image by trying each key one by one. Therefore, as long as the key space of the encryption algorithm is large enough, it can effectively resist the brutal attack. In this algorithm, the key includes the initial values of piecewise linear map, SHA-384, and 2D-LSCM, and its key space is about 2^442^. Therefore, the key space of the algorithm is large enough. It is difficult to find the initial key used by the encrypting step and by the brutal attack using the existing computer, so it can effectively resist the brutal attack.

### 4.2. Key Sensitivity Analysis

**Figure 11:**
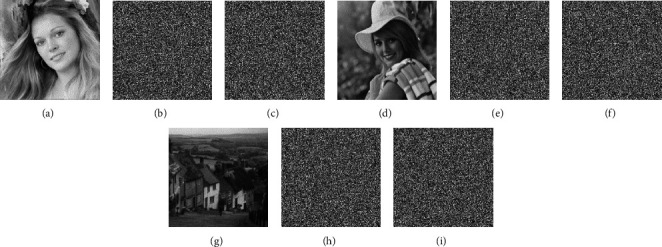
Recovered images using a slightly changed key. (a) Face. (b) Encrypted face. (c) Decrypted face. (d) Elaine. (e) Decrypted elaine. (f) Decrypted elaine. (g) Hill. (h) Encrypted hill. (i) Decrypted hill.

In response to hacker attacks, a good initial key sensitivity can make the encryption system more resistant. In the case of keeping other parameters unchanged, changing a certain key to a very small value, then try to decrypt and simulate the results, and we can get the key sensitivity analysis of the encryption system. This analysis changes the key *c*_*i*_^0^ to (*c*_*i*_^0^ + ∆*K*) and (*c*_*i*_^0^ + ∆*K*). To quantitatively analyze the key sensitivity, the ciphertext difference rate (CDR) analysis is performed on the encrypted images before and after the key change according to formula 11.  Figure 11 shows the results of decryption using the changed key, and Table 3 shows the CDR results. It shows that a small change in the key can cause the decrypted image to be different from the original image [35].

(11) $$\begin{array}{c} \left\{\begin{array}{c} Y=C\left(I,K\right)\text{,}\\ Y_{1}=C\left(I,K+∆K\right)\text{,}\\ Y_{2}=C\left(I,K-∆K\right)\text{,}\\ Diff\left(A,B\right)=\overset{M-1}{\underset{i=0}{\sum }}\overset{N-1}{\underset{j=0}{\sum }}Diff\;p\left(A\left(i,j\right),B\left(i,j\right)\right),\\ Diff\;p\left(A\left(i,j\right),B\left(i,j\right)\right)=\left\{\begin{array}{c} 1,A\left(i,j\right)\neq B\left(i,j\right)\text{,}\\ 0,A\left(i,j\right)=B\left(i,j\right)\text{,} \end{array}\right.\\ CDR=\frac{Diff\left(Y,Y_{1}\right)+Diff\left(Y,Y_{2}\right)}{2M*N}*100\%\text{,} \end{array}\right. \end{array}$$

where *Y* represents the original decrypted image, and *Y*_1_ and *Y*_2_ represent two decrypted images encrypted with a slightly changed key. The *I* represents the original image, *K* represents a key, and *C* represents the encryption algorithm.

### 4.3. Histogram Analysis

**Figure 12:**
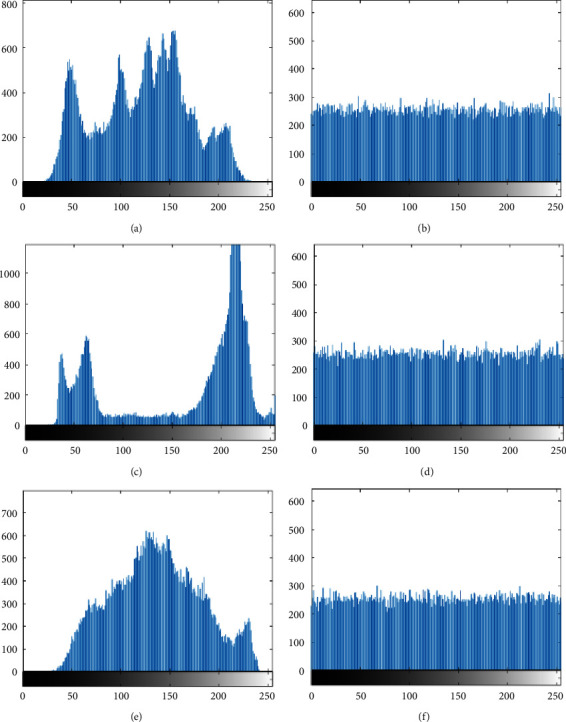
Histogram of image and encrypted image. (a) Lena histogram. (b) Encrypted Lena histogram. (c) Cameraman histogram. (d) Encrypted cameraman Histogram. (e) Elaine histogram. (f) Encrypted elaine histogram.

How many pixels appear in each gray value of the histogram can get very intuitive data, which directly reflects whether the distribution of image matrix elements is uniform or not. In the encryption algorithm, it is generally believed that the more average the encrypted result is, the noisier the distribution is, and the better the encryption effect is.  Figure 12 shows the histogram data comparison between the encrypted images and the original images in the simulation results of the proposed algorithm. From the histogram analysis, we can see that the pixel distribution of the original image has been effectively disrupted. The correlation between each pixel has also been broken, and its pixel distribution has achieved a very uniform and smooth effect.

The uniformity of the histogram is evaluated by the chi-square test by formula (12).  Table 4 shows the chi-square test results of the three groups of decrypted images of size 256*∗*256. It can be seen that the proposed encryption algorithm accepts the null hypothesis, and the *p*-value of the password images is greater than 0.05, indicating the consistency of the histogram samples. Therefore, it can be concluded that the proposed encryption algorithm can effectively resist statistical attacks [36].

(12) $$\begin{array}{c} x^{2}=\overset{M*N}{\underset{i=1}{\sum }}\frac{{\left(P\left(i\right)-T\left(i\right)\right)}^{2}}{T\left(i\right)}\text{,} \end{array}$$

where *P*(*i*) is the pixel value and *T*(*i*) is the theoretical value.

### 4.4. Correlation Analysis of Adjacent Elements

The correlation of adjacent elements of the encrypted image should be as small as possible, including horizontal correlation, vertical correlation, and diagonal correlation. When the correlation properties of adjacent elements are completely broken, it is difficult for attackers to break the ciphertext image through statistical analysis. We perform correlation analysis on Lena image of size 256*∗*256. The calculation of the correlation of adjacent elements [37] is shown in formula 13.  Table 5 shows the correlation calculation results of adjacent elements in all directions of the original image and the encrypted image.

*x* and *y* represent the gray values of two adjacent pixels in the image. We randomly selected 2500 pairs of pixels from all directions and compared them with the correlation of adjacent pixels in references [38–41]. The correlation between pixels in the calculation results is as follows:

(13) $$\begin{array}{c} \left\{\begin{array}{c} E\left(x\right)=\frac{1}{N}\overset{N}{\underset{i=1}{\sum }}x_{i}\text{,}\\ D\left(x\right)=\frac{1}{N}\overset{N}{\underset{i=1}{\sum }}{\left(x_{i}-E\left(x\right)\right)}^{2}\text{,}\\ \;Cov\left(x,y\right)=\frac{1}{N}\overset{N}{\underset{i=1}{\sum }}\left(x_{i}-E\left(x\right)\right)\left(y_{i}-E\left(y\right)\right)\text{,}\\ r_{xy}=\frac{Cov\left(x,y\right)}{\sqrt{D\left(x\right)}*\;\sqrt{D\left(y\right)}}\text{.} \end{array}\right. \end{array}$$

It can be seen from Table 5 that the encryption scheme breaks the correlation between adjacent pixels very well, and the encryption effect is better than that of these reference algorithm performances. Therefore, the encryption effect is very good and can effectively resist attacks.

### 4.5. Information Entropy and Local Entropy

Information entropy is an important index to measure the encryption effect of the encryption algorithm. The ideal value of information entropy of a good encryption algorithm should be close to 8. The calculation process of information entropy is shown in the following formula:

(14) $$\begin{array}{c} H\left(X\right)=-\overset{255}{\underset{i=0}{\sum }}P\left(i\right)\log_{2}\;P\left(i\right)\text{,} \end{array}$$

where *P*(*x*_*i*_) represents the probability of occurrence of gray value *x*_*i*_. If the entropy of the encrypted result is close to 8, the encryption effect of the system is effective.

The local information entropy measures the randomness of an image by calculating the average value of the information entropy over multiple nonoverlapping and randomly selected image blocks as a measure to describe the randomness of the test image [42]. We calculate the local information entropy of the image by the formula as follows:

(15) $$\begin{array}{c} \bar{H_{k,T_{B}}}\left(S\right)=\overset{k}{\underset{i=1}{\sum }}\frac{H\left(S_{i}\right)}{k}\text{,} \end{array}$$

where *T*_*B*_ is a randomly selected pixel and *k* is the number of randomly selected nonoverlapping image blocks with *T*_*B*_ pixels.

Global and local analysis of information entropy on Lena ciphertext image of size 256*∗*256.  Table 6 shows the information entropy and the local information entropy of the ciphertext image obtained by the proposed encryption algorithm and compared with other algorithms. It can be seen from Table 6 that the information entropy of the ciphertext image obtained by the proposed encryption scheme is close to 8. Although the local information entropy is significantly lower than the global information entropy, it is still close to 8. Therefore, the encryption scheme has a good ability against statistical analysis attack.

### 4.6. Differential Attack Analysis

NPCR and UACI are important indexes to evaluate the security of encryption algorithms when resisting differential attacks. The most point to resist differential attack is to compare the difference between two encrypted images. NPCR refers to the change rate of the number of pixels and UACI refers to the uniform average change intensity. The calculation process of the above two indexes is shown in formula (16). To measure the performance of the proposed encryption algorithm from a more secure perspective, Wu et al. proposed a comprehensive study of randomness tests including NPCR and UACI, and they focused on the theoretical critical NPCR and UACI values for encrypted images of different sizes. Tables 7 and 8 show the NPCR and UACI values for the three *α*-level hypothesis test scores for three different-size images, respectively [43].

Analyzing the sensitivity of the image encryption algorithm is analyzed by changing one pixel in Lena image of size 256*∗*256.  Table 9 shows the simulation results and compares them with other algorithms.

(16) $$\begin{array}{c} \left\{\begin{array}{c} NPCR=\overset{M}{\underset{i=1}{\sum }}\overset{N}{\underset{j=1}{\sum }}C\left(i,j\right)/M*N\;*\;100\%\text{,}\\ C\left(i,j\right)=\left\{\begin{array}{c} \;0,P_{1}\left(i,j\right)=P_{2}\left(i,j\right)\text{,}\\ \;1,P_{1}\left(i,j\right)\neq P_{2}\left(i,j\right)\text{,} \end{array}\right.\\ UACI=\overset{M}{\underset{i=1}{\sum }}\overset{N}{\underset{j=1}{\sum }}\left|P_{1}\left(i,j\right)-P_{2}\left(i,j\right)\right|/255*M*N\;*100\%\text{,} \end{array}\right. \end{array}$$

where *M* and *N* are the specifications of the image, *P*_1_ is the plaintext image, and *P*_2_ is the ciphertext image. The ideal value of NPCR and UACI should be 100% and 33%, respectively [43].

The results given in Table 9 all satisfy the three *α*-significance level tests of NPCR and UACI, indicating that the encrypted images generated by the proposed encryption algorithm are very different from the original images. Therefore, it can be considered that its encryption effect has good performance against differential attack.

### 4.7. Data Loss Attack Analysis

**Figure 13:**
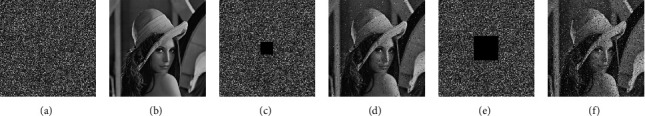
Encrypted image and its corresponding decrypted image after data loss. (a) Encrypted image. (b) Decrypted image. (c) 1/64 occlusion. (d) Decrypted image with 1/64 occlusion. (e) 1/16 occlusion. (f) Decrypted image with 1/16 occlusion.

A good encryption system should be able to recover the plaintext image as much as possible when it is attacked and lose data. Even if the ciphertext image loses part of the data when being attacked, the decrypted image obtained through the system should be able to express the main information of the original image. Data loss attack analysis means that in the simulation experiment, the pixels in a certain area of the encrypted image are destroyed artificially, then they are used as the input of the decrypted image for operation. Then the recovery degree of the decrypted image is analyzed and compared with the original image. We cut out 1/64 and 1/16 elements on the Lena ciphertext image of size 256*∗*256 to be analyzed and decrypt as shown in Figure 13. The correlation between the adjacent pixels in the decrypted image and the original image can be used as the quantitative analysis of the results, which are shown in Table 10. The calculation of the correlation of adjacent pixels is shown in formula (13). The peak signal-to-noise ratio (PSNR) can be used to measure the decryption recovery ability of the encrypted image [44, 45]. The calculation of PSNR is shown in formula (17), and the results are shown in Table 11. Through comparison, the algorithm can recover the information features of the original image effectively. Therefore, the proposed encryption system has excellent ability against data loss attack.

(17) $$\begin{array}{c} \left\{\begin{array}{c} PSNR=10*\;\log\frac{255^{2}}{MSE}\left(dB\right),\\ MSE=\frac{1}{M*N}\overset{M-1}{\underset{i=0}{\sum }}\overset{N-1}{\underset{j=0}{\sum }}{\left(I_{P}\left(i,j\right)-I_{C}\left(i,j\right)\right)}^{2}, \end{array}\right. \end{array}$$

where *I*_*P*_ and *I*_*C*_ are the plaintext image and ciphertext image, respectively.

### 4.8. Noise Attack Analysis

Image information in the transmission channel is usually subject to a variety of interference, such as pulse interference, resulting in some light and dark impurities in the ciphertext image matrix, which is called noise. The appearance of noise will destroy the original data in the image information, resulting in a certain degree of loss of the decrypted image information calculated by the receiver. We discuss the attack effect of salt and pepper noise on the encryption system. The attack ratio of pepper and salt noise represents the attack intensity of external interference on the image data, that is, the number of pepper and salt noise point replacement in every 100-pixel point. After adding salt and pepper noise with a certain attack intensity, the encrypted image is calculated by the decryption system, and the decrypted image is compared with the original image. The correlation of adjacent pixels is used as the reference index for quantitative analysis.

**Figure 14:**
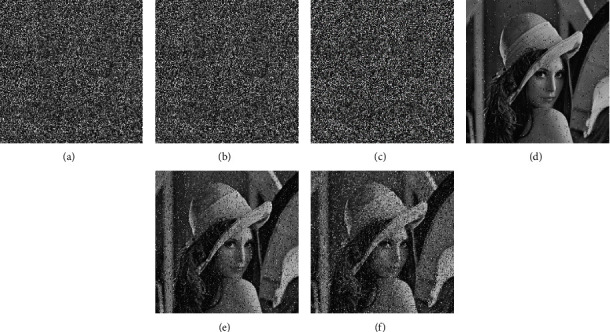
Encrypted image and its corresponding decrypted image after noise attack. (a) Attack ratio 1%. (b) Attack ratio 5%. (c) Attack ratio 10%. (d) Decrypted image with attack ratio 1%. (e) Decrypted image with attack ratio 4%. (f) Decrypted image with attack ratio 10%.

Figure 14 shows the simulation results of noise attack on Lena ciphertext image of size 256*∗*256. The correlation analysis of the adjacent pixels of the decrypted image is shown in Table 12. The PSNR results are shown in formula (17), and the results are shown in Table 11. It can be concluded that the encryption algorithm still retains a good ability against salt and pepper noise attacks.

### 4.9. NIST Test

We use pseudorandom sequences in the image encryption algorithm proposed in this paper. The randomness of the pseudorandom sequence will affect the security of the encryption system. So we need to test the randomness of the pseudorandom number sequences generated by the two chaotic systems. In this paper, we use the NIST test to examine the randomness of pseudorandom sequences. If the NIST test result *p* ∈ [0.01,1], the sequence is random.

The results are shown in Table 13. We can see that both the PWLCM system and the 2D-LSCM system passed all randomness tests.

### 4.10. Time Complexity Analysis

The time complexity of the encryption algorithm determines its calculation cost [46, 47]. A good encryption system should have a faster encryption speed. The number of running time required for the operation by a computer program can be expressed as a function *f*(*n*) whose independent variable is the input size *n*. Time complexity is the index used to estimate the operation time of the program. The time complexity of an algorithm is usually measured by *f*(*n*), which is called *O*(*f*(*n*)). The number of operations ignores the constant value and the coefficient of the highest power. Assuming that the number of pixels of the image to be encrypted is the input size *n*, the time complexity of the algorithm proposed in this paper is *O*(*n*^2^). Therefore, the algorithm has high efficiency and can realize image encryption quickly, which can be applied to practical applications.

### 4.11. Chosen Plaintext (Ciphertext) Attack Analysis

The cracker uses the assumed plaintext (ciphertext) to calculate the corresponding ciphertext (plaintext) through the encryption system, then obtains the encryption key by analyzing its characteristics, and finally cracks the original image, which is called the chosen plaintext (ciphertext) attack. In this attack simulation, the existing permutation-based encryption algorithm is easy to crack [48]. The attacker usually selects a pepper noise image with the same specification as the attacked image to obtain the intermediate key of the encryption algorithm. Then select a unique image that represents the position of a pixel to track this pixel, so as to obtain its position in the initial image, and finally achieve the purpose of cracking the entire initial image.

The encryption algorithm proposed in this paper has a strong defense against the chosen plaintext (ciphertext) attack. In the process of chaos initialization, the control parameters and initial values of the selected chaotic system are generated by the initial image, so the sensitivity is high enough. When the cracker attacks, because all the gray pixel values of the pepper noise image are 0, the chaotic sequence generated according to this algorithm will change greatly with the change of the original image. This kind of attack cannot track the change of any pixel position to crack the original image. Even if the cracker chooses the special image attack of non-noise, as long as the cracker cannot get the accurate initial value, he cannot get the chaotic sequence which is fed back and confused with the plaintext image. The attacker cannot track the transfer of the pixel position by assuming the image. Therefore, the encryption algorithm proposed in this paper can effectively resist the chosen plaintext (ciphertext) attack and ensure the security of important information.

## 5. Conclusion

In this paper, we propose an image encryption algorithm based on two-dimensional chaotic system and Josephus permutation. In this algorithm, the distribution of the pixel matrix is disturbed by Josephus traversal and gray curve to permute the pixel position. The binary value of the pixel is diffused by crossover operation and the ciphertext feedback, which breaks the strong correlation of image data. Experimental and simulation results show that the proposed encryption scheme has a large key space, high sensitivity to the encrypted image and the initial key, and the encryption results are good enough in various analysis indicators and have good defense ability against various attacks. Therefore, it can be considered that the proposed encryption scheme is better than the existing encryption algorithm, which makes up for the shortcomings of the traditional encryption algorithm, such as complex management, and slow computing speed, and can be used for image information encryption and information security protection.

## Figures and Tables

**Table 1:** Coding rules.

		0000
		0001
		0011
		0010
	000	0110
	001	0111

00	011	0101

01	010	0100

11	110	1100

10	111	1101
	101	1111
	100	1110
		1010
		1011
		1001
		1000

**Table 2:** Crossover operation.

*A*	1	0	1	1	1	0	1	0
*B*	0	1	0	0	0	1	0	1
Operator	1	0	0	1	0	1	1	0
*A*′	1	1	0	1	0	0	1	1
*B*′	0	0	1	0	1	1	0	0

**Table 3:** CDRs between cipher images with different keys.

Groups	Key value			CDR (%)
1	*c*_*i*_^0^ ± ∆*K*	*c*_2_^0^	*c*_3_^0^	99.62
2	*c*_1_^0^	*c*_2_^0^ ± ∆*K*	*c*_3_^0^	99.63
3	*c*_1_^0^	*c*_2_^0^	*c*_3_^0^ ± ∆*K*	99.61

**Table 4:** Histogram consistency assessment based on chi-square test.

	Lena	Cameraman	Elaine
*P*-value	265.22656	261.66406	257.21094
Decision	Passed	Passed	Passed

**Table 5:** Comparison correlation coefficient values of the Lena image.

	Horizontal	Vertical	Diagonal
Original image	0.9639	0.9361	0.9030
Encrypted image	−0.0013	−0.0008	−0.0017
Reference [38]	0.0090	0.0079	0.0032
Reference [39]	0.0011	0.0005	0.0016
Reference [40]	0.0008	-0.0019	-0.0016

**Table 6:** Comparative information entropy of the Lena image.

	Entropy	Local entropy
Original image	7.4532	7.4532
Encrypted image	7.9973	7.9053
Reference [38]	7.9975	7.9029
Reference [39]	7.9970	7.9017
Reference [40]	7.9972	7.9024

**Table 7:** Theoretical NPCR critical values for images of different sizes (%).

Image size	Result value	Theoretical NPCR critical values		
		0.05-level	0.01-level	0.001-level
128*∗*128	99.6094	99.5292	99.4960	99.4588
		Pass	Pass	Pass

256*∗*256	99.6117	99.5693	99.4690	99.4588
		Pass	Pass	Pass

512*∗*512	99.6334	99.5893	99.5810	99.5717
		Pass	Pass	Pass

**Table 8:** Theoretical UACI critical values for images of different sizes (%).

Image size	Result value	Theoretical UACI critical values		
		0.05-level	0.01-level	0.001-level
128*∗*128	33.6997	*U*_−0.05_^*∗*^ = 33.1012	*U*_−0.01_^*∗*^ = 32.9874	*U*_−0.001_^*∗*^ = 32.8552
		*U*_+0.05_^*∗*^= 33.8259	*U*_+0.01_^*∗*^ = 33.9397	*U*_+0.001_^*∗*^ = 34.0718
		Pass	Pass	Pass

256*∗*256	33.4570	*U*_−0.05_^*∗*^ = 33.2824	*U*_−0.01_^*∗*^ = 33.2255	*U*_−0.001_^*∗*^ = 33.1594
		*U*_+0.05_^*∗*^ = 33.6447	*U*_+0.01_^*∗*^ = 33.7016	*U*_+0.001_^*∗*^ = 33.7677
		Pass	Pass	Pass

512*∗*512	33.4073	*U*_−0.05_^*∗*^ = 33.3730	*U*_−0.01_^*∗*^ = 33.3445	*U*_−0.001_^*∗*^ = 33.3115
		*U*_+0.05_^*∗*^ = 33.5541	*U*_+0.01_^*∗*^ = 33.5826	*U*_+0.001_^*∗*^ = 33.6156
		Pass	Pass	Pass

**Table 9:** Differential attack analysis (%).

	NPCR	UACI
Simulation result	99.6117	33.4570
Reference [38]	99.6634	33.7112
Reference [39]	99.9950	34.1222
Reference [40]	99.6109	33.4783
Reference [41]	99.6100	33.3800

**Table 10:** Data loss attack analysis.

	Horizontal	Vertical	Diagonal
Original image	0.9639	0.9361	0.9030
1/64 occlusion	0.8344	0.8185	0.7883
1/16 occlusion	0.6039	0.5545	0.5383

**Table 11:** PSNR of data loss and noise attack analysis.

Attacks	Data loss		Pepper and salt noise		
	1/64	1/16	1%	5%	10%
PSNR (%)	24.83	18.26	23.08	17.30	12.10

**Table 12:** Pepper and salt noise analysis.

	Horizontal	Vertical	Diagonal
Original image	0.9639	0.9361	0.9030
Attack ratio 1%	0.8976	0.8493	0.8303
Attack ratio 5%	0.6288	0.6128	0.5998
Attack ratio 10%	0.4588	0.4289	0.4084

**Table 13:** NIST tests.

Test item	*p*-value		Result
	PWLCM	2D-LSCM	
Approximate entropy	0.2301	0.2669	Passed
Block frequency	0.6024	0.2133	Passed
Cumulative sums	0.3382	0.0232	Passed
FFt	0.1837	0.4503	Passed
Frequency	0.1402	0.3744	Passed
Linear complexity	0.5263	0.4089	Passed
Longest run	0.8891	0.9916	Passed
Nonoverlapping template	0.5628	0.0202	Passed
Overlapping template	0.2787	0.2787	Passed
Random excursions	0.8343	0.1474	Passed
Random excursions variant	0.2780	0.9987	Passed
Ranks	0.6845	0.1463	Passed
Runs	0.1514	0.2047	Passed
Serial test	0.3696	0.0269	Passed
Maurer's universal	0.6985	0.8084	Passed

## Data Availability

The data used to support the findings of this study are included within the article.
